# An Examination of the Diagnostic Utility of Ubiquitin-Specific Peptidase 6 (USP6) Rearrangement in Differentiating Nodular Fasciitis From Inflammatory Myofibroblastic Tumor: A Case Report

**DOI:** 10.7759/cureus.69995

**Published:** 2024-09-23

**Authors:** Bret-Ashleigh Coleman, Kelly M Frasier, William Farmer, Christopher B Harmon, Charles A Parrish

**Affiliations:** 1 Dermatology, Edward Via College of Osteopathic Medicine, Auburn, USA; 2 Dermatology, Northwell Health, New Hyde Park, USA; 3 Dermatology, Surgical Dermatology Group, Vestavia Hills, USA; 4 Dermatopathology, DermLab, Birmingham, USA

**Keywords:** benign neoplasm, inflammatory myofibroblastic tumors, nodular fasciitis, soft-tissue sarcomas, usp6 rearrangement

## Abstract

Nodular fasciitis (NF) is a benign yet diagnostically challenging mesenchymal myofibroblastic proliferation that often mimics the histological features of inflammatory myofibroblastic tumors (IMTs) and soft tissue sarcomas. The overlap in histopathological appearance, compounded by the variability in immunohistochemical (IHC) staining, frequently leads to diagnostic uncertainty. In this report, we present a case of a rapidly expanding lesion on the left medial mandible, ultimately diagnosed as NF. Molecular analysis through fluorescence in situ hybridization (FISH) identified a ubiquitin-specific peptidase 6 (USP6; 17p13.2) gene rearrangement, a distinctive marker of NF, which played a critical role in confirming the diagnosis. IHC analysis, including negative staining for cytokeratin and ALK, further helped differentiate this benign entity from other IMTs and malignancies, highlighting the importance of combining molecular diagnostics with traditional histopathological techniques to ensure accurate classification and avoid misdiagnosis.

## Introduction

Nodular fasciitis (NF) is a unique pathological entity that closely mimics more aggressive neoplasms, such as sarcomas, both clinically and histologically. This benign mesenchymal proliferation, characterized by rapid growth and a tendency for spontaneous regression, often poses significant diagnostic challenges due to its alarming presentation. The tumor predominantly involves the proliferation of myofibroblasts and fibroblasts interspersed with varying quantities of collagen and mucin, creating a cellular environment that can be mistaken for malignancy. The anecdotal association of NF with preceding trauma further complicates its clinical course, as this trauma-history link has been inconsistently reported but remains a recurring feature. During its initial growth phase, NF typically exhibits an aggressive infiltrative behavior, with a high mitotic index, heightened cellularity, and associated pain, all of which contribute to its resemblance to soft tissue sarcomas [1]. This rapid expansion and cellular proliferation heighten clinical suspicion, often leading to misdiagnosis and unnecessary concern regarding malignancy.

Histologically, NF presents a spectrum of features that overlap with malignant neoplasms. It is composed of spindle-shaped myofibroblasts, often with varying degrees of collagen deposition and myxoid changes. The cellular architecture can appear chaotic and infiltrative, and without deeper molecular analysis, the rapid mitotic activity and cellular density may mimic the histopathological patterns of sarcomas. Due to these nonspecific characteristics, diagnosing NF based solely on traditional histological and immunohistochemical (IHC) evaluations can be fraught with difficulties. Historically, pathologists have relied on morphology as the cornerstone of diagnosis, but these methods alone have often resulted in significant diagnostic ambiguity [2]. IHC staining, while useful in some settings, tends to be non-specific in NF cases, offering little clarity in distinguishing benign from malignant processes. 

However, advances in molecular pathology have provided new tools to aid in the differentiation of NF from more aggressive myofibroblastic tumors. One of the key breakthroughs in recent years has been the identification of genetic rearrangements, specifically involving ubiquitin-specific peptidase 6 (USP6). Fluorescence in situ hybridization (FISH) analysis has shown that USP6 gene rearrangements are a recurrent feature in NF, which provides a reliable molecular marker that can assist in differentiating NF from other entities within the differential diagnosis, such as sarcomas or inflammatory myofibroblastic tumors (IMTs) [2]. This chromosomal aberration, now recognized as a hallmark of NF, significantly enhances diagnostic accuracy, reducing the likelihood of misdiagnosis and the subsequent overtreatment that could result from assuming a malignant pathology.

In this case report, we present a case of NF, in which the diagnosis was confirmed through the identification of USP6 rearrangement via FISH analysis. This case highlights the critical role of molecular diagnostics in distinguishing NF from malignant or more aggressive benign mimickers, underscoring the importance of integrating chromosomal analysis into the diagnostic workflow for soft tissue lesions. The ability to definitively identify NF through genetic analysis not only helps in ensuring accurate diagnosis but also aids in alleviating patient anxiety and guiding appropriate therapeutic strategies.

## Case presentation

**Figure 1:**
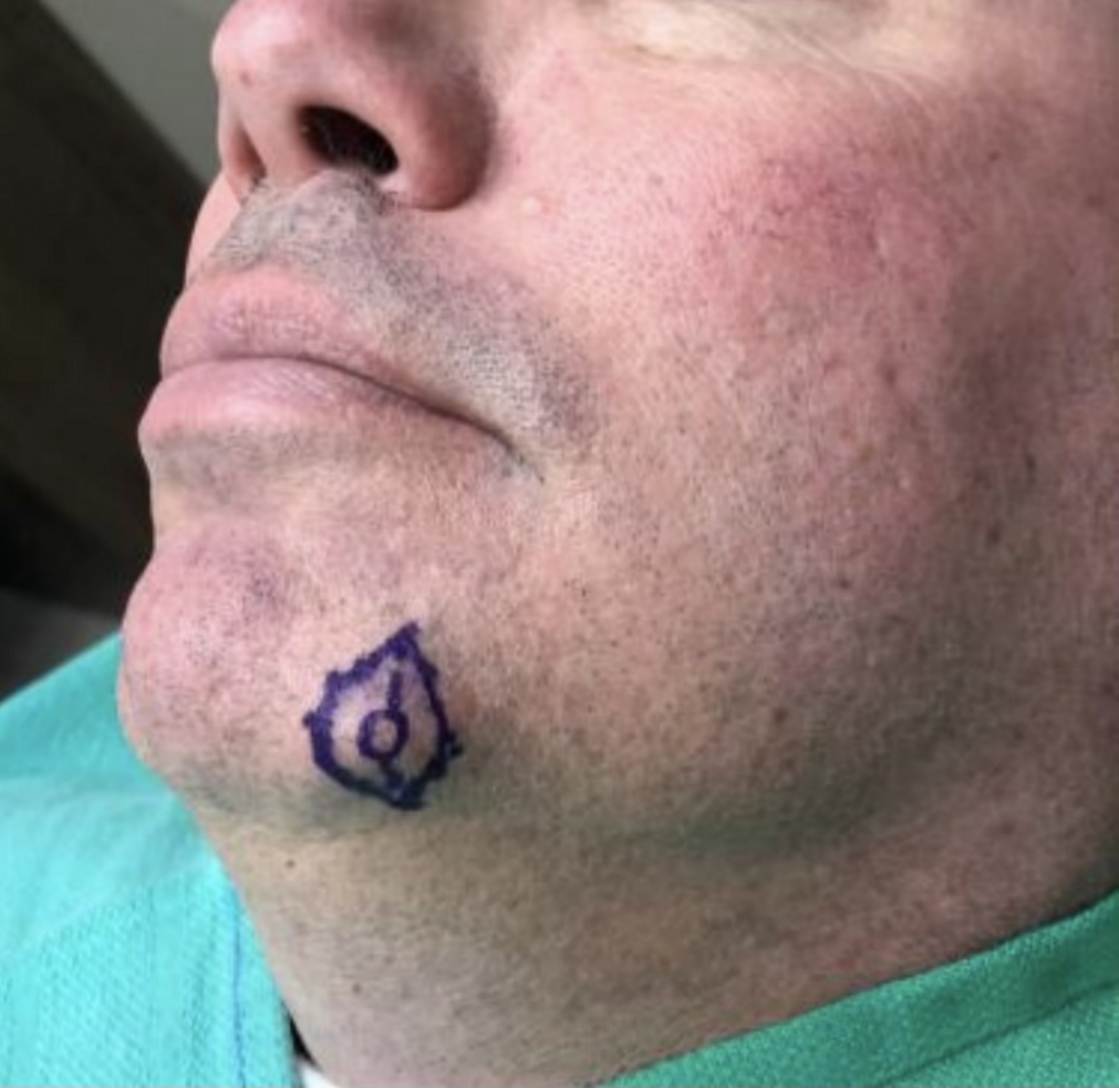
0.7 × 0.6 cm nodule on the left medial mandible

A 58-year-old male presented with a rapidly developing subcutaneous nodule measuring 0.7 × 0.6 cm, located on the left medial mandible (Figure 1). The patient reported no associated symptoms such as pain, tenderness, or functional impairment, and there was no significant history of trauma to the region. The nodule had emerged suddenly and expanded over the course of one month, prompting clinical evaluation. Due to its atypical rapid growth, an excisional biopsy was performed to rule out malignancy and identify the precise etiology of the lesion.

**Figure 2:**
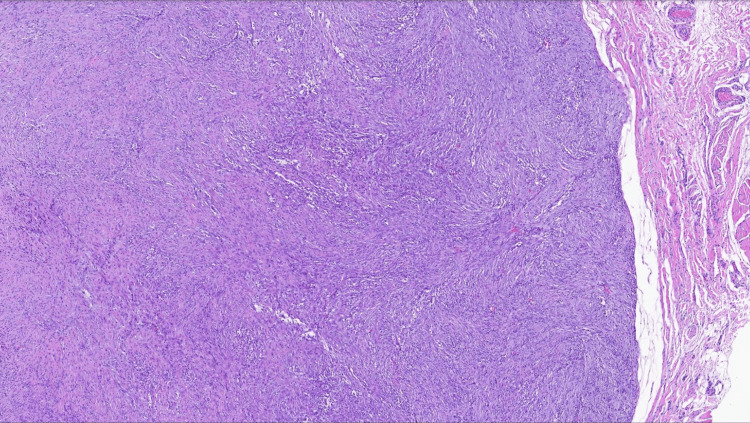
Nodular and ovoid tumor with a small focus on the plexiform arrangement

**Figure 3:**
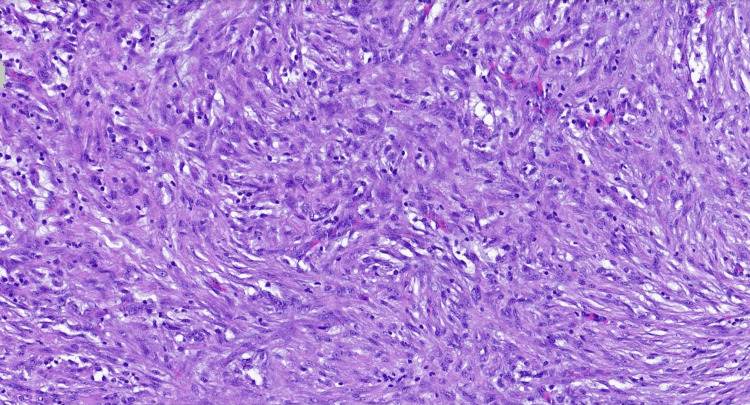
Nodule proper containing smallish spindled and fusiform cells in the stroma that vary from myxoid to collagenous with small pools of mucin occasionally present

Histopathological analysis of the excised nodule revealed scattered inflammatory infiltrates interspersed with small, spindled, and fusiform cells, consistent with reactive myofibroblastic proliferation. The spindle cells, with their elongated nuclei and eosinophilic cytoplasm, were distributed within a loose myxoid matrix, a feature commonly associated with NF (Figures 2, 3). The cellular composition was relatively homogeneous, with no evidence of nuclear pleomorphism, necrosis, or aberrant mitotic figures that would raise suspicion for a malignant sarcoma. However, given the infiltrative growth pattern and rapid onset, a more definitive diagnosis required further IHC characterization and molecular testing.

IHC staining was performed to narrow down the differential diagnosis. The spindle cells exhibited positivity for calponin and smooth muscle actin (SMA), markers associated with myofibroblastic differentiation, further supporting the diagnosis of a benign reactive process. Notably, the lesion tested negative for SOX10, desmin, CD34, ERG, ALK, and cytokeratin, which effectively ruled out other entities such as neural tumors, desmoid-type fibromatosis, vascular neoplasms, and IMTs. The absence of ALK positivity was particularly significant, as ALK rearrangements are a hallmark of IMTs, which would otherwise represent a key differential diagnosis. The exclusion of these markers helped to significantly narrow the diagnostic possibilities.

To confirm the diagnosis, FISH analysis was employed, focusing on the USP6 gene locus at 17p13.2. The analysis revealed a distinct USP6 rearrangement, a genetic abnormality that is highly characteristic of NF. The identification of this chromosomal aberration further solidified the diagnosis, distinguishing the lesion from other spindle cell neoplasms, such as soft tissue sarcomas and IMTs, which do not exhibit this specific rearrangement. Taken together, the histological features, IHC profile, and molecular findings confirmed the benign nature of the lesion, allowing for a definitive diagnosis of NF.

## Discussion

NF is a benign, rapidly growing, and self-limiting pseudosarcomatous tumor that often raises suspicion for malignancy due to its aggressive clinical presentation [3]. Primarily composed of vascular and fibroblastic proliferation, NF commonly occurs in adults in areas such as the head and neck, upper extremities, and trunk. Although typically asymptomatic, its fast growth rate, often reaching its final size within a few weeks, mimics malignant processes. This growth pattern, combined with its infiltrative appearance, contributes to frequent misdiagnosis as a malignant soft tissue sarcoma or an IMT. As a result, clear diagnostic tools are essential to prevent unnecessary and potentially harmful overtreatment, such as extensive surgical excision or even chemotherapy and radiation, which are inappropriate for this benign condition.

Historically, the diagnosis of NF was largely reliant on its histological morphology. Key features include a proliferation of spindle cells arranged in short S-shaped bundles within a myxoid stroma, often accompanied by extravasated erythrocytes [4]. The discohesive nature of the cells, often described as having a tissue culture or feathery appearance, is another characteristic finding. Additionally, osteoclast-like giant cells may be seen in some cases [5]. The histological appearance of NF can also evolve with time; newer lesions tend to exhibit a predominantly myxoid composition, while older lesions become more fibrotic. Despite these distinguishing features, the histopathological overlap with other soft tissue tumors, particularly IMTs and low-grade sarcomas, complicates the diagnostic process. The challenge lies in the fact that these histological patterns are not exclusive to NF, creating potential pitfalls when attempting to differentiate it from malignant entities.

In an attempt to enhance diagnostic accuracy, IHC staining has been explored as a complementary diagnostic tool. Commonly used markers for NF include SMA, muscle-specific actin, vimentin, and calponin, all of which highlight the myofibroblastic nature of the tumor. However, these stains are also frequently positive in IMTs, further complicating the diagnostic picture. For instance, SMA and calponin positivity are not unique to NF and can be observed in a variety of benign and malignant spindle cell tumors [5]. This overlapping immunophenotypic profile often limits the utility of IHC staining alone in distinguishing between NF and its malignant mimics. Consequently, the variability of staining patterns has led to the realization that more specific diagnostic criteria are needed to conclusively identify NF.

The advent of molecular diagnostics, specifically FISH analysis, has provided a significant breakthrough in the diagnosis of NF. A study conducted in 2011 demonstrated that USP6 gene rearrangements were present in approximately 92% of NF cases, marking this genetic abnormality as a highly reliable diagnostic marker [6]. USP6, located on chromosome 17p13, encodes a deubiquitinating enzyme involved in various cellular processes, including protein turnover, cell transformation, and inflammatory signaling [7]. The identification of this rearrangement has proven to be a critical differentiator, particularly in cases where histological and IHC findings are ambiguous. FISH analysis offers a level of specificity that histology and IHC alone cannot achieve, allowing for more accurate differentiation between NF and other spindle cell neoplasms, such as IMTs and sarcomas [8].

One of the most commonly identified fusion partners of USP6 is myosin heavy chain 9 (MYH9), located on chromosome 22q13 [9]. This fusion has been implicated in the pathogenesis of NF and provides further insight into the molecular mechanisms underlying this tumor. Other fusion partners, such as protein phosphatase 4 regulatory subunit 3 (PPP6R3), have also been identified, raising the possibility that certain molecular variants of NF may carry different biological behaviors. For example, a PPP6R3-USP6 fusion gene with amplification has been observed in rare cases of malignant NF, suggesting that this genetic alteration may confer malignant potential. While the majority of NF lesions remain benign, these findings emphasize the importance of molecular analysis in determining the biological nature of the tumor and guiding appropriate treatment strategies.

The potential for overtreatment in NF is a significant concern, given its rapid growth and high mitotic activity, which can be misinterpreted as signs of malignancy. Surgical excision is often pursued out of caution despite NF’s well-documented propensity for spontaneous resolution. In a study by Cloutier et al., three cases of NF resolved without intervention within three, four, and 14 weeks, with no residual disease at one-year follow-up [10]. This evidence supports the notion that, in certain cases, a conservative approach to NF may be warranted, particularly when the diagnosis is confirmed via FISH analysis or other molecular techniques. By recognizing the benign nature of the condition and its tendency to self-resolve, clinicians can avoid unnecessary surgeries and focus on patient monitoring instead.

While NF is a benign and self-limiting condition, its clinical and histological resemblance to malignant neoplasms necessitates a careful and thorough diagnostic approach. The integration of molecular diagnostics, particularly FISH analysis for USP6 rearrangements, has provided a powerful tool in distinguishing NF from its malignant mimics, offering a level of diagnostic precision that was previously unattainable with histology and IHC alone. This advancement not only helps prevent overtreatment but also alleviates patient anxiety by confirming the benign nature of the lesion. As molecular techniques continue to evolve, they will undoubtedly play an increasingly vital role in the accurate diagnosis and management of soft tissue tumors such as NF.

## Conclusions

NF presents a significant diagnostic challenge due to its rapid growth, high mitotic rate, and histological resemblance to malignant tumors such as soft tissue sarcomas. Clinically, its aggressive appearance and rapid expansion often raise suspicion of malignancy, especially given its infiltrative nature and the presence of pain. Histologically, NF is characterized by spindle-shaped myofibroblasts within a myxoid or collagenous stroma, and its cellularity and mitotic activity often mimic malignant features, leading to potential misdiagnosis. While traditional diagnostic approaches have relied on histological and IHC analysis, these methods have proven inconsistent due to NF’s variable presentation and the non-specific nature of commonly used markers, such as SMA and calponin. IHC stains may assist in excluding other entities like IMTs or sarcomas, but this alone often fails to definitively diagnose NF. Recent advancements in molecular diagnostics, particularly the identification of recurrent USP6 gene rearrangements via FISH analysis, have dramatically improved diagnostic accuracy. These USP6 rearrangements, present in approximately 92% of NF cases, have emerged as a reliable molecular marker that can distinguish NF from other spindle cell tumors. The use of FISH analysis to detect USP6 rearrangements has become an essential tool in the differential diagnosis of NF, providing clarity in cases where histological and IHC findings are inconclusive. This molecular approach not only aids in distinguishing NF from its malignant mimics but also prevents overtreatment and alleviates patient anxiety by confirming the benign nature of the condition, underscoring the importance of integrating genetic testing into the diagnostic workflow for soft tissue tumors.
